# Vector capacity of *Anopheles sinensis* in malaria outbreak areas of central China

**DOI:** 10.1186/1756-3305-5-136

**Published:** 2012-07-09

**Authors:** Jia-Yun Pan, Shui-Sen Zhou, Xiang Zheng, Fang Huang, Duo-Quan Wang, Yu-Zu Shen, Yun-Pu Su, Guang-Chao Zhou, Feng Liu, Jing-Jing Jiang

**Affiliations:** 1 National Institute of Parasitic Diseases, Chinese Center for Disease Control and Prevention, Shanghai, 200025, People’s Republic of China; 2 WHO Collaborating Centre for Malaria, Schistosomiasis and Filariasis, Key Laboratory of Parasite & Vector Biology, Ministry of Health, Shanghai, 200025, People’s Republic of China; 3 Anhui Center for Disease Control and Prevention, Wuhu, 241000, People’s Republic of China; 4 Henan Center for Disease Control and Prevention, Zhengzhou, 450016, People’s Republic of China; 5 Yuangcheng Center for Disease Control and Prevention, Yuangchen, Henan province, 450000, People’s Republic of China; 6 Yongqiao District Center for Disease Control and Prevention, Shuzhou, Anhui province, 241000, People’s Republic of China

**Keywords:** Vector capacity, *Anopheles sinensis*, Outbreak, Malaria, *Plasmodium vivax*, China

## Abstract

**Background:**

Both falciparum and vivax malaria were historically prevalent in China with high incidence. With the control efforts, the annual incidence in the whole country has reduced to 0.0001% except in some areas in the southern borders after 2000. Despite this, the re-emergence or outbreak of malaria was unavoidable in central China during 2005–2007. In order to understand the role of the vector in the transmission of malaria during the outbreak period, the vector capacity of *An. sinensis* in Huanghuai valley of central China was investigated.

**Findings:**

The study was undertaken in two sites, namely Huaiyuan county of Anhui province and Yongcheng county of Henan province. In each county, malaria cases were recorded for recent years, and transmission risk factors for each study village including anti-mosquito facilities and total number of livestock were recorded by visiting each household in the study sites. The specimens of mosquitoes were collected in two villages, and population density and species in each study site were recorded after the identification of different species, and the blood-fed mosquitoes were tested by ring precipitation test. Finally, various indicators were calculated to estimate vector capacity or dynamics, including mosquito biting rate (MBR), human blood index (HBI), and the parous rates (M). Finally, the vector capacity, as an important indicator of malaria transmission to predict the potential recurrence of malaria, was estimated and compared in each study site.

About 93.0% of 80 households in Huaiyuan and 89.3% of 192 households in Yongcheng had anti-mosquito facilities. No cattle or pigs were found, only less than 10 sheep were found in each study village. A total of 94 and 107 *Anopheles* spp. mosquitos were captured in two study sites, respectively, and all of *An. sinensis* were morphologically identified. It was found that mosquito blood-feeding peak was between 9:00 pm and 12:00 pm. Man biting rate of *An. sinensis* was 6.0957 and 5.8621 (mosquitoes/people/night) estimated by using half-night human bait trap method and full-capture method, respectively. Human blood indexes (HBI) were 0.6667 (6/9) and 0.6429 (18/28), and man-biting habits were 0.2667 and 0.2572 in two sites, respectively. Therefore, the expectation of infective life and vector capacity of *An. sinensis* was 0.3649-0.4761 and 0.5502-0.7740, respectively, in Huanhuai valley of central China where the outbreak occurred, which is much higher than that in the previous years without malaria outbreak.

**Conclusions:**

This study suggests that vivax malaria outbreak in Huanhuai valley is highly related to the enhancement in vector capacity of *An. sinensis* for *P. vivax*, which is attributed to the local residents’ habits and the remarkable drop in the number of large livestock leading to disappearance of traditional biological barriers.

## Background

Malaria is one of the most important parasitic diseases in People’s Republic of China [1-3]. Historically, a higher incidence of malaria, caused by infection of both *Plasmodium vivax* and *P. falciparum*, was observed among human populations in central China, such as Anhui, Henan, Hubei, Shangdong and Jiangsu provinces, located in Huanghuai valley [2,4]. Two big epidemics of malaria occurred at the beginning of the 1960s and the 1970s, of which the incidence rate was 1.55% and 2.96%, respectively [5]. Following the great control efforts, the incidences of both species of malaria had reduced remarkably. For instance, only 0.1% to 0.0017% of annual malaria incidence was reported during the 1980s and the 1990s in the country, respectively [6]. While, the incidence of vivax malaria transmitted by *Anopheles sinensis* was increased after 2000, due to the social and environment changes in macro scenario, such as population mobility increase due to the development of the economy, global warming, leading to the longer transmission season for *P. vivax*, and so on [4,7-10]. About 86% of malaria cases in the country were located in the Huanghuai valley of central China where the predominant vector mosquito was *An. sinensis*[2,11,12]. In this region, most of the outbreaks that occurred were localized at village level or township level of 4 provinces in central China, including Anhui, Henan, Hubei and Jiangsu provinces [13,14]. Previous studies in central China have shown that vector capacity is one of most important factors contributing to the transmission of vivax malaria, which could accelerate re-emergence of malaria outbreaks [2,15-18], but only a few relevant field-epidemiological investigations on the re-emergence of vivax malaria have been reported in China as well as in the Greater Mekong Subregion [19,20].

China launched the National Malaria Elimination Program (NMEP) in 2010, with its goal to eliminate malaria in China by 2020 [21,22]. In the NMEP, vector control is one of the important components in rapid response to the malaria transmission in outbreak foci as well as to improve the efficiency of case management, which has been promoted by both World Health Organization (WHO) and Roll Back Malaria Partnership (RBM) for reduction of malaria transmission [23,24]. One of the most difficult issues in the elimination process is to have real-time surveillance and response systems to monitor the changes of transmission patterns in order to guide the elimination efforts in the high risk areas [8]. It is believed that transmission intensity of human malaria is highly dependent on the vector capacity and competence of local mosquitoes. Recent research aimed at understanding the relationship between vector capacity and the transmission patterns of malaria is of significant concern [25,26]. Therefore, it is important to understand the changes of *An. sinensis* vector capacity in the high risk areas or in outbreak foci. But few investigations have explored the changes in vector capacity of *An. sinensis* at the micro settings, although it is believed that the vector capacity of mosquitoes is a good indicator to assess the transmission level of vivax malaria [26-28]. It is worthwhile understanding the role of the anopheles mosquitoes in the transmission of vivax malaria in Huanghuai valley during outbreaks, which will guide the formulation of elimination strategy and establishment of surveillance and response systems in the NMEP in China.

The investigation on vector capacity of *An. sinensis* in two outbreak areas of Anhui and Henan provinces was carried out during summer of 2007 when vivax malaria sporadic outbreaks were still taking place, in order to clearly understand the change patterns for vector capacity of *An. sinensis* which will promote the surveillance-response strategy [26].

## Methods

### Area and time of investigation

**Figure 1:**
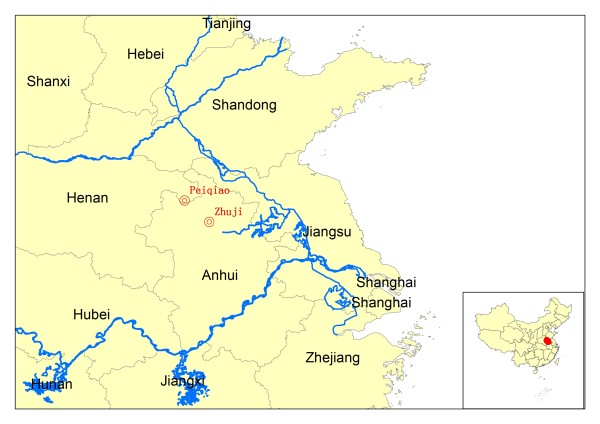
**Location of the two study sites.** One study site was located in the Zhao village, Zhuji township, Huaiyuan county (33°14’N and 116°51’E), Anhui province (site A), the other one was located in Pengguanyao village, Peiqiao township, Yongcheng county (33°48’N and 116°12’E), Henan province.

The study was undertaken in two sites, including Zhao village, Zhuji township, Huaiyuan county (33°14’N and 116°51’E), Anhui province, and Pengguanyao village, Peiqiao township, Yongcheng county (33°48’N and 116°12’E), Henan province (Figure 1). Both counties are located in the plain region, surrounded with rice pads, and planted with crops, such as beans, rice etc., and chemical compounds are rarely used as insecticides. The drainage system is not adequate, facilitated with more open water around villages in Summer time, thus providing more suitable habitats for the development of *An. sinensis* larvae.

The investigation was carried out at two time points, e.g. early August and late August, 2007, respectively, with an interval of 10 days, during the peak transmission period of vivax malaria and its sporadic outbreaks which still occurred in the study region. Half-night mosquito biting experiments were undertaken twice in Huaiyuan county and Yongcheng county, respectively. Full-capture method was used to collect *Anopheles* spp. mosquito specimens in the early morning inside and outside of mosquito-nets in human living rooms, as well as in livestock rooms in the study sites.

### Malaria incidence and risk factors

All reported data on malaria cases recorded by the Center for Disease Control and Prevention in each county was collected, and the malaria incidence in each study county was estimated in 2007 and compared with reported data from the region in previous years. Malaria transmission-related risk factors for each study site, including usage of anti-mosquitofacilities and total number of livestock, were recorded by visiting each household of the study villages.

### Vector capacity

The specimens of mosquito were collected in two villages, and population density and species in each study site were recorded after the identification of different species, based on morphology of mosquitoes, and the saturated mosquitoes were tested by ring precipitation test [29]. Finally, various indicators were calculated to estimate vector capacity, including mosquito biting rate (MBR), human blood index (HBI), and the parous rates (M) [30,31] using the following details of methodology.

The vector capacity was estimated from the calculation of three indices [32]. Firstly, the investigation on mosquito biting rate was conducted by two approaches. One approach is the half-night bite rate, which was carried out during 19:30 pm to 24:00 pm by the human attraction method, this was carried out 4 times to capture as many anopheles mosquitoes as possible [29]. Briefly, a person was inside the half-opened mosquito-net, and *Anopheles* spp. mosquitoes were continuously collected over the time period [12]. The number of *An. sinensis* collected was recorded. The half-night man biting rate was calculated by following formula:

(1) $$\text{Half-night man biting rate}\;(\text{No}.\text{mosquitoes}/\text{people})\;\text{=}\frac{\text{number of mosquitoes captured inside and outside the net}}{\text{number of people for bait trap inside net}}$$

The other approach used to investigate mosquito biting rate was performed in the same villages using a full-capture method [33]. Briefly, *Anopheles* spp. mosquitoes were searched for and captured in the early morning during 6:00 am to 7:00 am. All *Anopheles* spp. mosquitoes were captured inside each mosquito-net in houses, and barnyards in the investigation village, covering a population of 300–450 people for each village. The number of *An. sinensis* captured was recorded, and the mosquito man-biting rate per night was calculated by the following formula:

(2) $$\text{Man biting density inside net whole night}\;\left(\text{No}.\text{mosquitoes}/\text{people }\right)=\frac{\text{the number of mosquitoes captured from nets}}{\text{the number of people in the nets surveyed}}$$

And then,

Man-biting rate (ma) (No. mosquitoes/ people/night) = half-night man biting rate + Man biting density inside net whole night.

Secondly, the human blood index (HBI) was estimated using the method of ring precipitation test [29]. All stomach blood specimens were examined from the captured *Anopheles* spp. mosquitoes engorged with blood after these mosquitoes had been morphologically identified. A serologic ring precipitation test was conducted to estimate the HBI, both HBI and man-biting habit (a) were calculated by using the following formula [33]:

(3) $$\text{HBI }=\frac{\text{No}.\text{mosquitoes of the human blood}}{\text{No}.\text{mosquitoes saturated}}$$

(4) $$\text{Man}-\text{biting habit }\left(\text{a}\right)=\frac{\text{HBI}}{\text{Gonotrophic cycle of}\;An.sinensis\left(\text{x}=2.5\text{ days}\right)}$$

Thirdly, the parous rate (M) was estimated. Briefly, egg-laying mosquitoes were distinguished by dissecting ovaries to see the morpho-rami of ovarial trachea, and x stands for the period in days from human biting to laying eggs, which will be easily considered as the gonotrophic cycle period, e.g. *An. sinensis* will take about 2.5 days to finish the gonotrophic cycle. Then, the parous rate (M) and daily survival rate (p = M^1/x^) were estimated. Given the local average temperatures and the sporgenous cycle (n) of 10 days [31,34], we estimated the expectation of infective life of *An. sinensis* (p^n^/–lnP).

Finally, the vector capacity (C) [35], an important indicator and measure of malaria transmission predicting recurrence of transmission [29,35-37], was estimated by using the following formula [29]:

(5) $$\text{Vector capacity }\left(\text{C}\right)=\frac{{\text{ma}}^{2}{\text{P}}^{\text{n}}}{-\text{ lnP}}$$

Where P is survival rate of mosquitoes, n is the mean days for reproduction of *Plasmodium* sporozoites in mosquitoes under the local temperature.

### Ethical considerations

The study protocol was approved by the institutional review board of the National Institute of Parasitic Diseases, Chinese Center for Disease Control and Prevention in Shanghai. The objectives, procedures, and potential risks of the mosquito biting experiment using the human attraction method were carefully explained to each participant. Interested individuals provided written informed consent in person before participation in the study.

## Results

### The malaria incidence and risk factors

The incidence of malaria in 2007 were 9.1/10^4^ (1167/1287000) and 22.2/10^4^ (2883/1297000) in Huaiyuan county, Anhui province and Yongcheng county, Henan province, respectively.

A total of 343 residents from 86 households in Huaiyuan, and 860 residents from 215 households in Yongcheng were visited. About 93.0% of the households (80/86) in Huaiyuan and 89.3% of the households (192/215) in Yongcheng had anti-mosquito facilities, such as mosquito nets, screened windows, screened doors and mosquito repellent. On average, about 1.3 (51/39) and 1.7 (219/129) people were used for one mosquito-net, and their mosquito-net coverage rates per household were 45.34% and 60.0% in Huaiyuan and Yongcheng, respectively. Neither cattle nor pigs were found in the study villages of two counties, only 9 and 7 sheep free-roaming were found in Huaiyuan and Yongcheng, respectively.

### Vector capacity

#### *Anopheles populations and its blood-sucking behavior*

**Figure 2:**
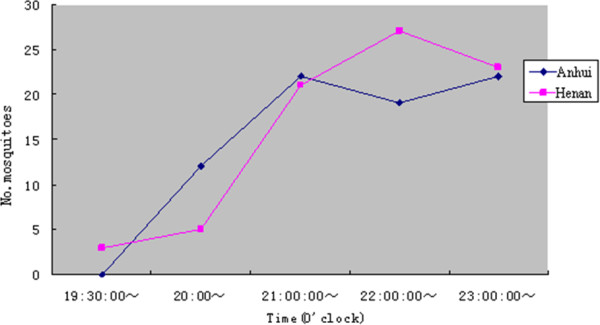
**Patterns of blood feeding activities of *Anopheles sinesis* at night.** Both curves indicating patterns of mosquito blood feeding activities from Anhui (blue line) and Henan (pink line) provinces.

The half-night human bait trap was performed twice in Huaiyuan county and Yongcheng county, respectively. A total of 201 *Anopheles* spp. mosquitoes were captured in study sites, among them 94 were found in Huaiyuan and 107 were collected in Yongcheng. Morphological analysis showed that they all belonged to species of *An.sinensis*. Results from the half-night human bait trap showed that *An. sinensis* activity for taking blood meals occurred from 7:30 pm to 12:00 pm, and the blood-feeding peak was between 9:00 pm and 12:00 pm (Figure 2).

**Figure 3:**
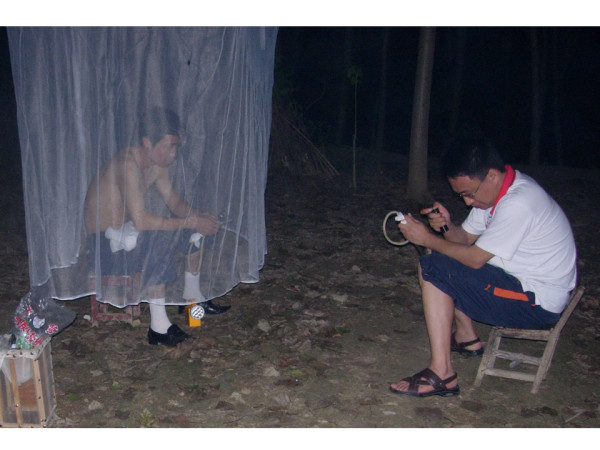
A photograph of *Anopheles sinesis* mosquito capture at night, using half-night human bait trap method inside mosquito net.

The mosquito specimens were collected by the full-capture method 3 times in total in the study sites. A total of 16 and 3 *An. sinensis* mosquitoes were obtained from 39 mosquito-nets and 3 livestock rooms in Huaiyuan county, respectively. 16 and 12 *An. sinensis* mosquitoes were collected from 42 mosquito-nets and 3 livestock rooms in Yongcheng, respectively. It was estimated that the man biting rate from the study sites was 6.0957 (mosquitoes/people/night) and 5.8621 (mosquitoes/people/night) by using the half-night human bait trap method and full-capture method, respectively (Table 1, Figure 3).

**Table 1:** Man-biting rate estimation in study sites

**Location**	**Bait method**	**Number of bait**	**Number of Capture**	Half-night bite rate *	**Biting rate in the mosquito nets§**	**Man-biting rate#**
Huaiyuan, Anhui province	Human bait outdoor	13	75	5.7692	0.3265(16/49)	6.0957
Yongcheng, Henan province	Human bait indoor and outdoor	14	79	5.6429	0.2192(16/73)	5.8621

Note: * Half-night bite rate was measured in mosquitoes/people; §Biting rate in the mosquito nets was measured in mosquitoes/people/night; and #Biting rate was measured in mosquito/people/night).

#### *Vector capacity*

A total of 47 *An. sinensis* mosquitoes were captured inside the houses, mosquito-nets and sheep yards in the study sites. Among them, 9 out of 19 *An. sinensis* mosquitoes engorged with blood were captured in Huaiyuan, and 28 out of 28 *An. sinensis* mosquitoes engorged with blood were captured in Yongcheng. Ring precipitation tests showed that HBI of these two study sites were 0.6667(6/9)and 0.6429(18/28), thus the man-biting habits (a) were 0.2667 and 0.2572 in Huaiyuan and Yongcheng, respectively.

A total of 93 *An.sinensis* were captured and dissected for examination of ovaries. The parous rates (M) were 57.14% (28/49) and 54.55% (24/44) from Huaiyuan and Yongcheng, respectively. Given the local average temperatures, the sporgenous cycle of *An. sinensis* was 10 days and the gonotrophic cycle was 2.5 days [38], we estimated that the expectations of infective life of *An. sinensis* were 0.4761 and 0.3649 from Huaiyuan and Yongcheng, respectively. Therefore, the vectorial capacities of *An. sinensis* in Huaiyuan and Yongcheng were 0.7740 and 0.5502, respectively (Table 2). When compared with the vector capacity of *An. sinensis* from the reported data in the previous years (1984–1994), the three indicators including the man-biting habit, human blood index and vector capacity were changed in consistency with the fluctuations of malaria incidence during the same period. The significant changes in man-biting habit reflected that the number of livestock as sources of infection changed during the study period as well, indicating that this change in pattern is closely related to the reduction of livestock numbers in the study sites.

**Table 2:** Comparison of vector capacity of *An. sinensis* between outbreak time period and non outbreak time period in areas where only species of *An. sinensis* served as vectors of vivax malaria

**Time (Location)**	**Incidence rate (%)**	**Man-biting rate (ma)**	**Human blood index (HBI)**	**Man-biting habit (a)**	**Expectation infective life (p**^**n**^/-lnp)*	**Vector capacity (C)Ma × a × (p**^**n**^**/-lnp)**
1984§	0.02	20.8	0.0300	0.0120	0.2424	0.0605
1994#	0.01	4.7	0.128	0.051	0.7275	0.1744
2007 (Huaiyuan)	0.091	6.0957	0.600	0.2667	0.4761	0.7740
2007 (Yongcheng)	0.222	5.8621	0.666	0.2572	0.3649	0.5502

*: Expectation of infective life was measured in days. §: Reported data in Huanghuai valley, China. #: Reported data in Henan provinces, China.

## Discussion

Vector control has been one of the important components for the malaria control program leading to elimination [39]. There are various methods of vector control that are used in blocking the transmission of *P. vivax*[28]*.* While the level of *P. vivax* transmission is significantly related to two main factors, such as vector capacity and the number of infectious sources or human cases [25]. When the environmental conditions are appropriate, malaria outbreaks could occur, involving large numbers of human cases [4,40].

In terms of infectious sources, human cases infected with *P. vivax* have been reduced significantly in the Huanghuai valley of China in the new millennium, although it was more than 10 million cases per year in the 1950s. In particular, following the large-scale efforts of the NMCP in China since the 1970s, malaria incidence in the areas where *An. sinensis* was the only vector had been reduced significantly to a low level in the 1990s. According to the national data on malaria transmission, the average annual incidences of vivax malaria in 1998 in Anhui and Henan province with *An. sinensis* being the only vector, were below 1/10^5^ and 0.03/10^5^, respectively. However, the incidence of vivax malaria had a resurgence in Huanghuai valley after 2003 [4,41]. For example, the annual incidence of malaria from 2004 to 2006 was 101.22/10^5^, 95.38/10^5^ and 90.67/10^5^ in Anhui province, and was 12.29/10^5^, 54.99/10^5^ and 222.82/10^5^ in Henan province, respectively, which were significantly higher than those recorded in the 1990s [42]. Serological investigation of malaria antibodies in these provinces in 2006 and 2007 showed that about 87.83% of the positive samples were asymptomatic cases [43], indicating that existing control measures were failing to protect these populations, since then the infection source of vivax malaria has been accumulating and increasing significantly.

For the vector capacity, human blood index (HBI) is an important and quantitative indicator, which showed the contact relationship between mosquitoes and humans [17]. HBI of the areas where *An. sinensis* was present was about 5% in the 1990s, which was attributed to the reduction in the incidence of vivax malaria in the region [44]. Reviewing the data during the 1990s, the number of pigs and farm cattle reared by each family increased, which caused the mosquitoes to feed more on blood from livestock instead of human blood. But since the development of the economy and popularity of agricultural mechanization, the farmers no longer keep pigs in their houses, and the number of farm cattle has also been greatly reduced. The increased probability of *An. sinensis* feeding on human blood indicates that the mosquitoes change their behavior caused by changes in number of livestock [45,46]. In addition, our study also revealed that the HBIs in Huaiyuan and Yongcheng were 0.6667 and 0.6429, and the HBI values were more than 12 times higher than that in the historical records. Results in the study also indicated that the increasing HBI value was due to the changes of human activities or living customs [25,47].

It has been observed that the local villagers generally used mosquito-nets and mosquito-repellent incense when asleep, and most families have screened-doors, and/or screened-windows. Only 7 out of 81 mosquito nets had *Anopheles* spp. mosquitoes inside nets in the morning of the investigation, which indicated that the chance of contact between mosquitoes and humans might occur. However, the mosquito-biting was inevitable because the villagers had a habit of enjoying cool outdoor fresh air before midnight in the Summer time [2].

The average man-biting rate observed in the 1990s in the areas where *An. sinensis* was the only vector was 2.36 [48], however, the rates in the study sites were 6.0957 and 5.8621 according to this investigation, which is more than 2.5 times higher than before. From the history data, the average vector capacity was 0.331 in the 1990s in Huanghuai valley where *An. sinensis* was mostly predominately distributed [48], and was 0.1686 during 1996–1998 in Henan province [2,38]. However, our findings showed that the vector capacity was 0.7740 and 0.5502 in Anhui and Henan province, respectively, about 2.3 and 1.7 times higher than that in the 1990s, and were 4.6 and 3.3 times higher than that in Henan during 1996–1998, respectively. All these results demonstrated that the ability of *An. sinensis* to transmit *P. vivax* had been obviously enhanced; therefore, more effective and practical control measures need to be implemented in Huanghuai valley, particularly in the mosquito control and reduction of the residual carriers with *P. vivax*. Our findings illustrated that the vector capacity is a good indicator to assess the malaria transmission capability, which contributes to changes in transmission patterns of vivax malaria.

## Conclusions

This study suggests that vivax malaria outbreaks in Huanghuai valley is highly related to the enhancement in transmission ability for *P. vivax* through *An. sinensis*, which is attributed to the habits of local residents and the remarkable drop in the number of large livestock leading to disappearance of traditional biological barriers.

## Competing interests

The authors declare that they have no competing interests.

## Authors’ contributions

Conceived and designed the experiments: JYP, SSZ, XZ, FH. Performed the experiments: XZ, FH, DQW, MZS, YPS, GCZ, JJJ. Analyzed the data: JYP, SSZ, XZ. Contributed reagents/materials/analysis tools: JYP, SSZ, XZ, FH. Wrote the paper: JYP, SSZ, XZ. All authors read and approved the final version of the manuscript.

## Financial support

The study was funded through the National S & T Mayor Project (Grant no.2008ZX10004-011, 2012ZX10004-22).

## References

[B1] Ning X Qin L Jinchuan Y Jiuping Y Xintian L Surveillance of risk factors from imported cases of falciparum malaria in Sichuan, China Southeast Asian J Trop Med Public Health 1999 30 235 239 10774684

[B2] Zhou SS Huang F Wang JJ Zhang SS Su YP Tang LH Geographical, meteorological and vectorial factors related to malaria re-emergence in Huang-Huai River of central China Malaria J 2010 9 337 10.1186/1475-2875-9-337 PMC3003275 21092326

[B3] Zhou SS Zhang SS Wang JJ Zheng X Huang F Li WD Xu X Zhang HW Spatial correlation between malaria cases and water-bodies in Anopheles sinensis dominated areas of Huang-Huai plain China Parasit Vectors 2012 5 106 22650153 10.1186/1756-3305-5-106 PMC3414776

[B4] Sleigh AC Liu XL Jackson S Li P Shang LY Resurgence of vivax malaria in Henan Province, China Bull World Health Organ 1998 76 265 270 9744246 PMC2305715

[B5] Qian HL Tang LH The achievement and the outlook of 50 years malaria prevention and cure work of China Chin J Epi 2000 21 225 227 in Chinese

[B6] Tang L Progress in malaria control in China Chin Med J (Engl) 2000 113 89 92 11775219

[B7] Hui FM Xu B Chen ZW Cheng X Liang L Huang HB Fang LQ Yang H Zhou HN Yang HL Zhou XN Cao WC Gong P Spatio-temporal distribution of malaria in Yunnan Province, China AmJTrop Med Hyg 2009 81 503 509 19706922

[B8] Yang GJ Gao Q Zhou SS Malone JB McCarroll JC Tanner M Vounatsou P Bergquist R Utzinger J Zhou XN Mapping and predicting malaria transmission in the People’s Republic of China, using integrated biology-driven and statistical models Geospat Health 2010 5 11 22 21080317 10.4081/gh.2010.183

[B9] Huang F Zhou S Zhang S Zhang H Li W Meteorological factors-based spatio-temporal mapping and predicting malaria in central China AmJTrop Med Hyg 2011 85 560 567 10.4269/ajtmh.2011.11-0156 PMC3163885 21896823

[B10] Wen L Li C Lin M Yuan Z Huo D Li S Wang Y Chu C Jia R Song H Spatio-temporal analysis of malaria incidence at the village level in a malaria-endemic area in Hainan China Malaria J 2011 10 88 10.1186/1475-2875-10-88 PMC3094226 21492475

[B11] Zhou SS Tang LH Sheng HF Wang Y [Malaria situation in the People’ s Republic of China in 2004] Zhongguo Ji Sheng Chong Xue Yu Ji Sheng Chong Bing Za Zhi 2006 24 1 3 in Chinese 16866130

[B12] Liu XB Liu QY Guo YH Jiang JY Ren DS Zhou GC Zheng CJ Zhang Y Liu JL Li ZF Chen Y Li HS Morton LC Li HZ Li Q Gu WD The abundance and host-seeking behavior of culicine species (Diptera: Culicidae) and Anopheles sinensis in Yongcheng city People’s Republic of China Parasit Vectors 2011 4 221 10.1186/1756-3305-4-221 22115320 PMC3267684

[B13] Zhou SS Wang Y Fang W Tang LH [Malaria situation in the People’s Republic Of China in 2007] Zhongguo Ji Sheng Chong Xue Yu Ji Sheng Chong Bing Za Zhi 2008 26 401 403 in Chinese 19288908

[B14] Zhou SS Tang LH Sheng HF [Malaria situation in the People’s Republic of China in 2003] Zhongguo Ji Sheng Chong Xue Yu Ji Sheng Chong Bing Za Zhi 2005 23 385 387 in Chinese 16566200

[B15] Gu ZC Shang LY Chen JS Zheng X Su YJ Li AM Liu H Luo MZ Qian HL Tang LH [The role of Anopheles anthropophagus in malaria transmission in in Xinyang City of Henan Province] Zhongguo Ji Sheng Chong Xue Yu Ji Sheng Chong Bing Za Zhi 2001 19 221 224 in Chinese 12571970

[B16] Qian H Tang L Cheng Y Yang B [Preliminary estimation of malaria transmission potential in areas where Anopheles sinensis is the only vector] Zhongguo Ji Sheng Chong Xue Yu Ji Sheng Chong Bing Za Zhi 1994 12 265 267 in Chinese 7720199

[B17] Liu C [Comparative studies on the role of Anopheles anthropophagus and Anopheles sinensis in malaria transmission in China] Zhonghua Liu Xing Bing Xue Za Zhi 1990 11 360 363 in Chinese 2276188

[B18] Liu XB Liu QY Guo YH Jiang JY Ren DS Zhou GC Zheng CJ Liu JL Chen Y Li HS Li HZ Li Q Random repeated cross sectional study on breeding site characterization of Anopheles sinensis larvae in distinct villages of Yongcheng City People’s Republic of China Parasit Vectors 2012 5 58 10.1186/1756-3305-5-58 22444032 PMC3323357

[B19] Paaijmans KP Blanford S Chan BH Thomas MB Warmer temperatures reduce the vectorial capacity of malaria mosquitoes Biol Lett 2012 8 465 468 10.1098/rsbl.2011.1075 22188673 PMC3367745

[B20] Sinka ME Bangs MJ Manguin S Chareonviriyaphap T Patil AP Temperley WH Gething PW Elyazar IR Kabaria CW Harbach RE Hay SI The dominant Anopheles vectors of human malaria in the Asia-Pacific region: occurrence data, distribution maps and bionomic precis Parasit Vectors 2011 4 89 10.1186/1756-3305-4-89 21612587 PMC3127851

[B21] Zhou SS Wang Y Xia ZG [Malaria situation in the People’s Republic Of China in 2009] Zhongguo Ji Sheng Chong Xue Yu Ji Sheng Chong Bing Za Zhi 2011 29 1 3 in Chinese 21823314

[B22] Qi G [Opportunities and challenges of malaria elimination in China] Zhongguo Xue Xi Chong Bing Fang Zhi Za Zhi 2011 23 347 349 in Chinese 22164839

[B23] WHO Malaria vector control and personal protection: report of a WHO study group 2006 World Health Organization, Geneva 16623084

[B24] RBM Key facts, figures and strategies: the Global Malaria Action Plan 2008 Roll Back Malaria Partnership, http://www.rbm.who.int/toolbox/tool_GMAP.html

[B25] Cohuet A Harris C Robert V Fontenille D Evolutionary forces on Anopheles: what makes a malaria vector? Trends Parasitol 2010 26 130 136 10.1016/j.pt.2009.12.001 20056485

[B26] Garrett-Jones C Prognosis for Interruption of Malaria Transmission through Assessment of the Mosquito’s Vectorial Capacity Nature 1964 204 1173 1175 10.1038/2041173a0 14268587

[B27] Paaijmans KP Blanford S Chan BH 2011 Warmer temperatures reduce the vectorial capacity of malaria mosquitoes. Biol Lett, Thomas MB 10.1098/rsbl.2011.1075 PMC3367745 22188673

[B28] Cui L Yan G Sattabongkot J Chen B Cao Y Fan Q Parker D Sirichaisinthop J Su XZ Yang H Yang Z Wang B Zhou G Challenges and prospects for malaria elimination in the Greater Mekong Subregion Acta Trop 2011 121 240 245 21515238 10.1016/j.actatropica.2011.04.006 PMC3155744

[B29] Garrett-Jones C Shidrawi GR Malaria vectorial capacity of a population of Anopheles gambiae: an exercise in epidemiological entomology Bull World Health Organ 1969 40 531 545 5306719 PMC2556109

[B30] Vythilingam I Phetsouvanh R Keokenchanh K Yengmala V Vanisaveth V Phompida S Hakim SL The prevalence of Anopheles (Diptera: Culicidae) mosquitoes in Sekong Province, Lao PDR in relation to malaria transmission Trop Med Int Health 2003 8 525 535 10.1046/j.1365-3156.2003.01052.x 12791058

[B31] Ijumba JN Mosha FW Lindsay SW Malaria transmission risk variations derived from different agricultural practices in an irrigated area of northern Tanzania Med Vet Entomol 2002 16 28 38 10.1046/j.0269-283x.2002.00337.x 11963979

[B32] Ree HI Hwang UW Lee IY Kim TE Daily survival and human blood index of Anopheles sinensis, the vector species of malaria in Korea J Am Mosq Control Assoc 2001 17 67 72 11345422

[B33] Luo MZ Zheng X Chen CY Comparison of three survey methods for testing blood preference of Anopheles sinensis in Wuxue City Chin J Parasit Dis Contrl 1994 7 219 221 in Chinese

[B34] Kere NK Arabola A Bakote’e B Qalo O Burkot TR Webber RH Southgate BA Permethrin-impregnated bednets are more effective than DDT house-spraying to control malaria in Solomon Islands Med Vet Entomol 1996 10 145 148 10.1111/j.1365-2915.1996.tb00720.x 8744706

[B35] Molineaux L Dietz K Thomas A Further epidemiological evaluation of a malaria model Bull World Health Organ 1978 56 565 571 365384 PMC2395644

[B36] Dietz K Molineaux L Thomas A A malaria model tested in the African savannah Bull World Health Organ 1974 50 347 357 4613512 PMC2481186

[B37] Khan AQ Talibi SA Epidemiological assessment of malaria transmission in an endemic area of East Pakistan and the significance of congenital immunity Bull World Health Organ 1972 46 783 792 4538539 PMC2480888

[B38] Qu CZ Su TZ Wang MY Vectorial capacity of malaria transmission of Anopheles sinensis in Zhengzhou in nature J Henan Med Uni 2000 35 394 396 in Chinese

[B39] Control TmCGoV A research agenda for malaria eradication: vector control PLoS Med 2011 8 e1000401 21311587 10.1371/journal.pmed.1000401 PMC3026704

[B40] Tyagi BK A review of the emergence of Plasmodium falciparum-dominated malaria in irrigated areas of the Thar Desert, India Acta Trop 2004 89 227 239 10.1016/j.actatropica.2003.09.016 14732244

[B41] Liu XZ Xu BL [Malaria situation and evaluation on the control effect in Henan Province during 1990–2005] Zhongguo Ji Sheng Chong Xue Yu Ji Sheng Chong Bing Za Zhi 2006 24 226 229 in Chinese 17094630

[B42] Qian HL Malaria Situation in the People’s Republic of China in 1993 Chin J Parasitol Parasitic Dis 1994 12 161 164 in Chinese 7867146

[B43] Zheng X Tang LH Gu ZC Zhu TH Shi WQ Jiang WK Zhou SS Pan B Lin RX [Morphology and habits of An. anthropophagus and its role in malaria transmission in Hengqin Island of Zhuhai City] Zhongguo Ji Sheng Chong Xue Yu Ji Sheng Chong Bing Za Zhi 2007 25 488 491 in Chinese 18441899

[B44] Qian HL Deng D Guan DH Investigation and quantitative analysis of the components of vectorial capacity of Anopheles sinensis Chin J Parasltol Parasit Dis 1984 2 3 in Chinese 6467539

[B45] Habtewold T Prior A Torr SJ Gibson G Could insecticide-treated cattle reduce Afrotropical malaria transmission? Effects of deltamethrin-treated Zebu on Anopheles arabiensis behaviour and survival in Ethiopia Med Vet Entomol 2004 18 408 417 10.1111/j.0269-283X.2004.00525.x 15642008

[B46] Wu KC Chen WJ Wang ZG Cai XZ Deng D Hu LK Liu ZY Zhu WG Guan DH Jiang WK [Studies on distribution and behavior of Anopheles minimus and its role of malaria transmission in Hainan Province at present] Zhongguo Ji Sheng Chong Xue Yu Ji Sheng Chong Bing Za Zhi 1993 11 120 123 in Chinese 8174214

[B47] Hancock PA Thomas MB Godfray HC An age-structured model to evaluate the potential of novel malaria-control interventions: a case study of fungal biopesticide sprays Proc Biol Sci 2009 276 71 80 10.1098/rspb.2008.0689 18765347 PMC2614244

[B48] Qian HL Tang LH Tang LY Preliminary estimation on the critical value of man biting rate and vectorial capacity of Anopheles sinensis Practical Preventire Med 1996 3 1 2 in Chinese

